# Association between parenting and non-suicidal self-injury among adolescents in Yunnan, China: a cross-sectional survey

**DOI:** 10.7717/peerj.10493

**Published:** 2020-12-07

**Authors:** Yi Liu, Yuanyuan Xiao, Hailiang Ran, Xingting He, Linling Jiang, TianLan Wang, Run-xu Yang, Xiufeng Xu, Guangya Yang, Jin Lu

**Affiliations:** 1 National Health Commission Key Laboratory of Drug Addiction Medicine, Psychiatric Department, First Affiliated Hospital of Kunming Medical University, Kunming, China; 2 Department of Epidemiology and Health Statistics, School of Public Health, Kunming Medical University, Kunming, China; 3 Lincang Psychiatric Hospital, Lincang, China

**Keywords:** NSSI, Adolescents, Rejection, Emotional warmth, Overprotection, Parenting, Repeated NSSI, Severe NSSI

## Abstract

**Background:**

Non-suicidal self-injury (NSSI) among adolescents is prevalent and its rate has increased in recent years worldwide. Previous studies had investigated the association between parenting and childhood NSSI, but little is known about the relationship between parental rearing and repetition and severity of NSSI. The aim of this study was to investigate associations of parenting with NSSI and its repetition and severity in a representative adolescent sample from southwestern China.

**Methods:**

In this cross-sectional study, a sample of 2,705 adolescents (F/M: 1,245/1,460; mean age: 13.4 ± 2.2 years) was recruited from 14 randomly selected schools in Lincang municipality, Yunnan province, China. A self-report questionnaire was used to collect data. The Adolescent Non-Suicidal Self-Injury Function Assessment Scale and the short Chinese Egna Minnen av Barndoms Uppfostran (s-EMBU-C) were used to evaluate NSSI behaviors and parenting style, respectively. Univariate and multivariate logistic regression models were adopted to examine association between parenting and NSSI.

**Results:**

Overall lifetime prevalence of NSSI was 47.1% (95% CI [36.2–58.0]), with self-cutting being the most common form (23.5% (95% CI [19.3–27.7])), followed by hitting hard objects (23.4% (95% CI [20.2–26.7])) and pulling hairs (20.9% (95% CI [18.8–22.6])). In multiple logistic regression analyses, NSSI was positively associated with high level of father’s rejection (OR: 1.32 (95% CI [1.01–1.72])), high level of mother’s rejection (OR: 1.76 (95% CI [1.46–2.13])), low level of mother’s emotional warmth (OR: 1.42 (95% CI [1.15–1.75])), and high level of mother’s overprotection (OR: 1.74 (95% CI [1.49–2.03])), repeated NSSI was positively associated with low level of father’s emotional warmth (OR: 1.39 (95% CI [1.10–1.75])) and high level of mother’s overprotection (OR: 1.79 (95% CI [1.33–2.41])), and severe NSSI was positively associated with low level of father’s emotional warmth (OR: 1.64 (95% CI [1.11–2.43])) and high level of mother’s rejection (OR: 2.16 (95% CI [1.71–2.71])).

**Conclusion:**

NSSI is common among adolescents in southwestern China. Negative parenting styles are associated with NSSI, repeated NSSI, and severe NSSI. The development of intervention measures for preventing or reducing NSSI among Chinese adolescents in school settings should consider parenting styles.

## Introduction

Non-suicidal self-injury (NSSI) is often defined as one’s deliberate, direct destruction or alteration of body tissue without suicidal ideation and for purposes not socially or culturally sanctioned (Lloyd-Richardson et al., 2020). The common ways of NSSI include self-cutting or slashing, self-burning, self-battery, scratching, biting, wound interference, and head banging (Swannell et al., 2014). In a multi-national study, the lifetime NSSI rate was 18% (Zetterqvist et al., 2020). NSSI is commonly seen in adolescents. In community samples of adolescents, the lifetime self-injury rate is 13–45% (Brunner et al., 2014; Jacobson & Gould, 2007; Nock, 2010), while in psychiatric samples of child and adolescents, the rate is as high as 50% (Plener et al., 2015). In China, two newly published studies reported that 33.7–51% of the community adolescents had NSSI (Hu et al., 2020a, 2020b). There is also evidence that the rate of NSSI in adolescents is still rising (Muehlenkamp et al., 2012). Many studies have shown that NSSI is a strong predictor of later completed suicide (Asarnow et al., 2011; Bryan et al., 2014; Cox et al., 2012; Goldstein et al., 2012; Whitlock et al., 2013). Therefore, adolescents’ NSSI deserves research attention.

Family environment plays an important role in shaping the behaviors of children and adolescents. According to Attachment Theory, the parent-child relationship is the primary relationship that a child has and the health of the relationship influences child’s social and emotional development (Bowlby, 1988; Brumariu, 2015; Ivbijaro et al., 2019). Parents are the primary agents of children’s socialization (Collins et al., 2000) and key contributors to youth health and development (Sze, Ba & Dai, 2020; Whitlock et al., 2018). Therefore, some parental characteristics have been associated with NSSI in adolescents (Arbuthnott & Lewis, 2015; Bureau et al., 2010). Poor parenting has been proved to be associated with increased risk of children’s later depression, anxiety, hostility, and social discomfort (Anosike, Isah & Igboeli, 2020; Leung, Kwok & Ling, 2016). On the other side, parents’ punishment and strict upbringing are important risk factors of NSSI (Martin et al., 2016). In Western countries, several studies have identified that negative parenting style is a risk factor for adolescents’ NSSI, while positive parenting style attenuates this risk (Van Lissa et al., 2019; Victor et al., 2019).

Examining association between parental rearing style and NSSI is of important public health significance, particularly in the design and development of intervention measures aiming at preventing or reducing NSSI among Chinese adolescents. In this cross-sectional study, we analyzed the association of parenting rearing style with NSSI in a large representative sample of adolescents in southwestern China.

## Materials and Methods

### Subjects

A cross-sectional survey was carried out in Lincang municipality, China, from December 1 to December 13, 2019. Lincang is located in Yunnan province, southwestern China, bordering Myanmar, and inhabited by 22 ethnic minority groups. We used a three-stage cluster sampling approach to recruit subjects. In the first stage, one district, Linxiang, was randomly chosen from all districts and counties in Lincang; In stage 2, we randomly chose 5 primary schools, 5 middle schools, and 4 high schools from the selected district; In stage 3, we randomly selected 1–2 classes from each grade of each selected school. Considering that only those aged 10 years and older are able to understand the concept of death and suicide (Mishara, 1999), our subjects were restricted to be students aged 10–17 years. We excluded students who had hearing disability, were severely ill and refused to participate.

Prior to the questionnaire survey, written informed consent has been obtained from both legal guardians and participants. The study protocol was reviewed and approved by the Ethics Review Board of the Third People’s Hospital of Lincang City (Ethical Application Ref.: No. 01, 2019).

### Procedures and assessments

A self-report questionnaire was used to collect data. The whole process of questionnaire survey was supervised by trained medical undergraduates and postgraduates. The questionnaire contained three parts, which measured socio-demographics, parenting style, and NSSI behaviors.

Non-suicidal self-injury behaviors were measured by Adolescent Non-Suicidal Self-Injury Function Assessment Scale (Zhang, 2015). It has been proved that the scale has good reliability and validity for assessing NSSI behaviors in Chinese adolescents (Zhang, 2015). Repeated NSSI was defined as reporting NSSI at least two times. Participants endorsed NSSI and rated the severity of NSSI as “moderate”, “severe”, or “extremely severe” were those having severe NSSI.

The short Chinese Egna Minnen av Barndoms Uppfostra (s-EMBU-C) was used to assess parenting style. This scale has 2 parts with each having 21 questions: father’s and mother’s rearing styles. Each part has three dimensions: rejection, emotional warmth, and overprotection. Total numbers of items of rejection, emotional warmth, and overprotection dimensions were 6, 7, and 8, respectively. These items adopt a Likert 4-point scoring method: “Never” (1 point), “Occasionally” (2 points), “Frequently” (3 points), “Always” (4 points). The higher dimension score suggests that parents are more likely to adopt the corresponding parenting style. The s-EMBU-C has satisfactory internal consistency and construct validity. The Cronbach’s α coefficients of dimensions of s-EMBU-C were 0.74–0.84 in the Chinese college students (Jiang et al., 2010).

### Statistical analysis

Descriptive statistics were used to delineate socio-demographic characteristics, NSSI, and parenting style. Univariate logistic regression models were used to assess the association between parenting style and NSSI, repeated NSSI, and severe NSSI. Multivariate binary logistic regression models were used to examine the associations between NSSI, repeated NSSI, and severe NSSI and parental style, controlling for all socio-demographic variables. The statistical significance level was set at less than 0.05, two-tailed. All analyses were conducted by using R software (Version 3.3.3; The R Foundation for Statistical Computing, Vienna, Austria). Since multiple-stage sampling was used in this study, the “survey” package of R software was used to adjust for the clustering effect.

## Results

### Sample characteristics and prevalence of NSSI

A total of 3,234 participants were identified, of whom 84 were excluded due to age ≥18 years and 445 were excluded due to missing data. Finally, 2,705 students were included into the analysis with a response rate was 83.6%.

Among the respondents, the mean age was 13.4 years with a standard deviation (SD) of 2.2, and 46% were boys. Other detailed socio-demographic characteristics were summarized in Table 1.

**Table 1:** Social demographics of participants (*N* = 2,705).

Characteristics	Mean/Median	Prop. (Count)	Standard error/IQR
General			
Age	13.42^§^		2.18^¶^
Sex: boys		46.0 (1245)	
Grade			
Primary school		33.9 (916)	
Middle school		34.4 (931)	
High school		31.7 (858)	
Only child		71.2 (1925)	
Marital status of parents			
In marriage		90.8 (2455)	
Other		9.2 (250)	
NSSI behaviors			
No		52.9 (1431)	
Yes		47.1 (1274)	
Repeated NSSI		65.2 (830)	
Severe NSSI		25.5 (324)	
Father’s rearing style score			
Rejection	7^†^		3.0^‡^
Emotional warmth	20^†^		8.0^‡^
Overprotection	16^†^		5.0^‡^
Mother’s rearing style score			
Rejection	8^†^		3.0^‡^
Emotional warmth	21^†^		7.5^‡^
Overprotection	17^†^		6.0^‡^

**Notes:**

§ Mean.

¶ Standard error.

† Median.

‡ Interquartile range.

The lifetime prevalence of NSSI was 47.1% (95% CI [36.2–58.0]). Among those who reported NSSI, 65.2% reported repeated NSSI and 25.5% reported severe NSSI.

**Figure 1:**
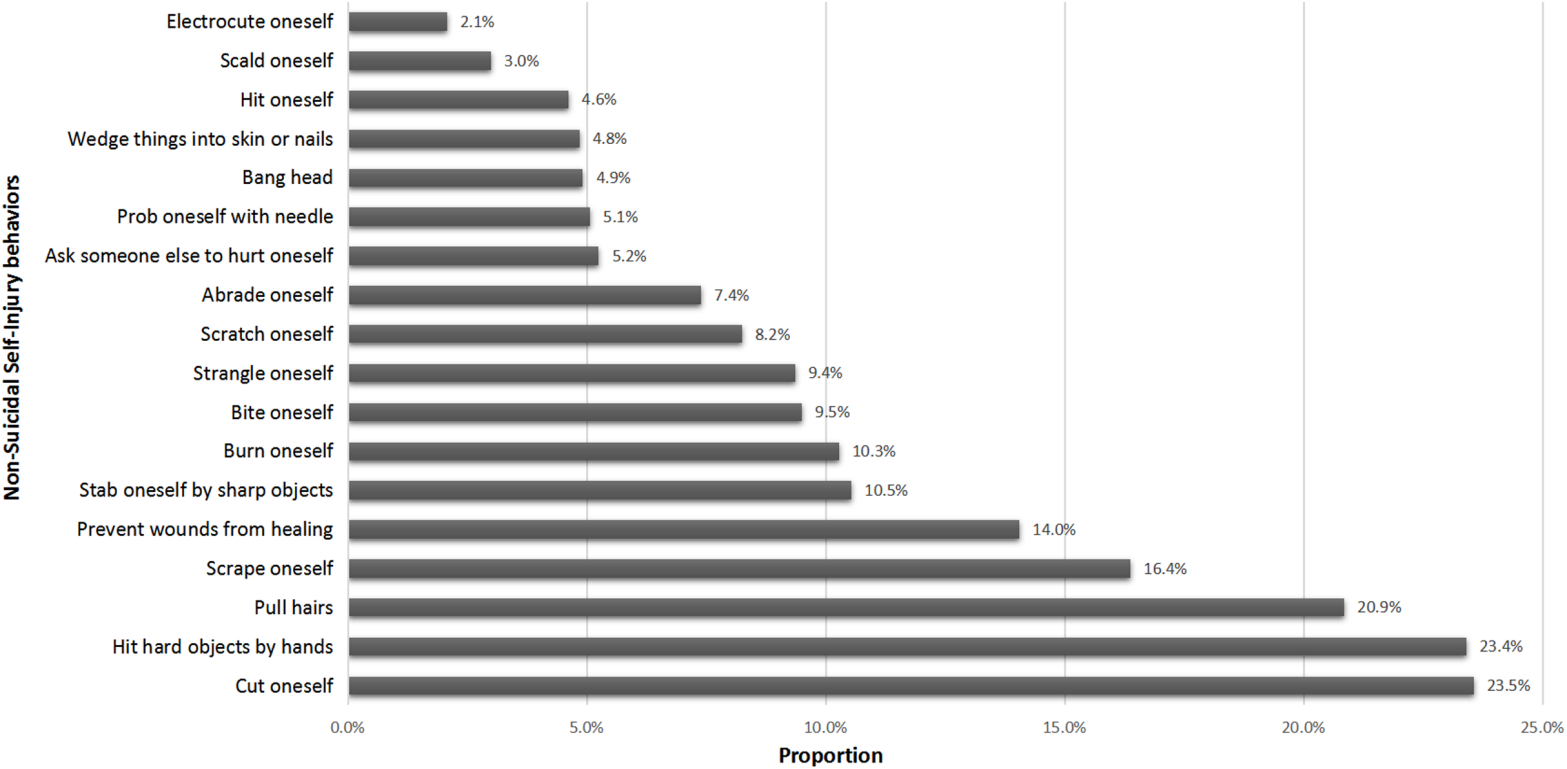
Proportion of different NSSI forms in all participants (*N* = 2,705).

The prevalence rates of different forms of NSSI behaviors were shown in Fig. 1. Self-cutting was the most prevalent (23.5% (95% CI [19.3–27.7])), followed by hitting hard objects by hands (23.4% (95% CI [20.2–27.2])) and pulling hairs (20.9% (95% CI [18.8–22.6])).

### Relationship between parenting style and NSSI, repeated NSSI, and severe NSSI

Results of univariate and multivariate logistic regression analyses on associations of parental style with NSSI, repeated NSSI, and severe NSSI were shown in Table 2. Each parenting style was dichotomized at its median score (“low” vs. “high”).

**Table 2:** Univariate and multivariate logistic regression models fitting results for NSSI, repeated NSSI, and severe NSSI.

Variables	NSSI	NSSI	Repeated NSSI	Repeated NSSI	Severe NSSI	Severe NSSI
	OR (95% CI)	Adjusted OR (95% CI)	OR (95% CI)	Adjusted OR (95% CI)	OR (95% CI)	Adjusted OR (95% CI)
Age: +1 year	1.28 [1.20–1.36]	1.11 [0.97–1.28]	1.16 [1.09–1.24]	1.05 [0.88–1.25]	0.97 [0.90–1.05]	1.02 [0.82–1.25]
Sex: Girls	1.34 [1.22–1.48]	1.46 [1.26–1.71]	0.83 [0.66–1.06]	0.78 [0.61–1.00]	0.98 [0.86–1.12]	1.03 [0.88–1.20]
Grade (Ref.: Primary school)						
Middle school	2.50 [1.50–4.17]	2.25 [1.11–4.58]	1.73 [1.15–2.61]	1.80 [1.08–3.01]	1.55 [1.14–2.10]	1.78 [0.96–3.32]
High school	3.66 [2.55–5.25]	2.63 [1.27–5.44]	2.30 [1.66–3.19]	2.16 [0.92–5.08]	0.89 [0.60–1.32]	1.04 [0.37–2.91]
Only child: Yes	0.79 [0.64–0.98]	1.06 [0.85–1.32]	1.43 [1.18–1.73]	1.56 [1.19–2.04]	1.01 [0.68–1.52]	1.24 [0.83–1.86]
Marital status of parents: Other	0.76 [0.66–0.89]	0.72 [0.55–0.94]	0.64 [0.40–1.02]	0.64 [0.36–1.14]	0.84 [0.58–1.21]	0.95 [0.62–1.45]
Father’s rearing style						
Rejection: High (Ref.: Low)	1.84 [1.46–2.31]	1.32 [1.01–1.72]	1.09 [0.81–1.45]	0.86 [0.66–1.13]	2.00 [1.38–2.90]	1.11 [0.75–1.65]
Emotional warmth: Low (Ref.: High)	1.79 [1.58–2.03]	1.21 [0.99–1.48]	1.37 [1.04–1.79]	1.39 [1.10–1.75]	1.37 [1.04–1.79]	1.64 [1.11–2.43]
Overprotection: High (Ref.: Low)	1.98 [1.67–2.33]	1.10 [0.94–1.28]	1.22 [1.03–1.45]	0.79 [0.61–1.03]	1.54 [1.19–1.98]	1.06 [0.78–1.44]
Mother’s rearing style						
Rejection: High (Ref.: Low)	2.16 [1.86–2.51]	1.76 [1.46–2.13]	1.33 [0.96–1.83]	1.39 [0.98–1.96]	2.70 [2.62–3.61]	2.16 [1.71–2.71]
Emotional warmth: Low (Ref.: High)	1.85 [1.71–2.01]	1.42 [1.16–1.75]	1.24 [0.94–1.63]	1.01 [0.75–1.37]	1.59 [1.10–2.31]	0.96 [0.57–1.63]
Overprotection: High (Ref.: Low)	2.30 [1.93–2.75]	1.74 [1.49–2.03]	1.61 [1.32–1.97]	1.79 [1.33–2.41]	1.75 [1.35–2.27]	1.34 [0.94–1.90]

In the adjustment analyses, high levels of rejection of father (OR: 1.32, 95% CI [1.01–1.72]) and mother (OR: 1.76, 95% CI [1.46–2.13]), low level of mother’s emotional warmth (OR: 1.85, 95% CI [1.71–2.01]) and high level of mother’s overprotection (OR: 1.74, 95% CI [1.49–2.03]), were significantly associated with NSSI. Low level of father’s emotional warmth (OR: 1.39, 95% CI [1.10–1.75]), and high level of mother’s overprotection of (OR: 1.79, 95% CI [1.33–2.41]) were significantly associated with repeated NSSI. Low level of father’s emotional warmth (OR: 1.64, 95% CI [1.11–2.43]) and high level of mother’s rejection (OR: 2.16, 95% CI [1.71–2.71]) were significantly associated with severe NSSI.

## Discussion

This study found that nearly a half (47.1%) of the participants reported NSSI in their lifetime. This prevalence estimate is considerably higher than those of previous studies in other countries (Brunner et al., 2014; Robinson, 2017; Zetterqvist et al., 2013), and also higher than prior studies in other regions of China (Mao, Li & Li, 2020; Zhang, Song & Wang, 2016). This prevalence difference may primarily due to the NSSI instrument used in our study, which is more comprehensive and detailed than those used in previous studies. Second, our sample was recruited from a multi-ethnic province in China, different cultural and geographic characteristics may also explain the high NSSI prevalence. Third, the high prevalence of NSSI may also be attributed to the high academic pressure and the high level of parent-child conflict of the study participants. Anyway, such a high prevalence suggests that NSSI is a public health challenge of adolescents in this municipality of south western China.

Attachment theory suggests that early childhood attachment experiences profoundly shape the social, cognitive and emotional development of adolescents, in which mothers and fathers play different roles (Bresin & Schoenleber, 2015). Previous studies also emphasized the roles of parent-child relationships on the development of NSSI among adolescents (Muehlenkamp et al., 2013; Taliaferro et al., 2020). Our study found the significant associations between parenting style and NSSI and its repetition and severity, which differ between fathers and mothers. These associations could be partly explained by the low parental emotional support associated with poor parental style, which increases the risk of NSSI in adolescents (Ammerman & Brown, 2018; Victor et al., 2019). The different associations by fathers and mothers are expected because, in general, mothers have greater chances to take part in the daily life of adolescents than fathers. Mothers, as the most important companion in the growth stage of the children, play a major role in children’s values and attitudes towards life and deaths, and therefore may influence the risk of NSSI of their children. Additionally, parental rejection might make adolescents feel upset and frustrated, and they might use NSSI to relieve unpleasant emotions.

Further analyses revealed that mother’s overprotection and father’s low emotional warmth were also associated with repeated NSSI. In the context of inadequate emotional support and stressful life events, adolescent might response in an ineffective manner such as NSSI (Nock, 2009). As a supporting case in point, a study showed that adolescents with repeated NSSI were not able to effectively cope with psychosocial stress (Klimes-Dougan et al., 2019). In addition, mother’s overprotection is related to children’s unhealthy self-conscious emotions, which has been associated with the presence of NSSI and frequent NSSI (Spitzen et al., 2020).

Another major finding from our adjustment analysis was that father’s emotional warmth and mother’s rejection were significantly associated with severe NSSI, suggesting that adolescents who perceived more emotional support from fathers and less rejection from mothers have less severe NSSI. These findings are consistent with previous studies (Wan et al., 2013; Xu et al., 2010).

Several limitations of the study should be considered. First, the study sample was chosen from a single city of Yunnan province only, thus our findings may not be generalized to adolescents of other parts of China. Second, this is a cross-sectional study, so longitudinal studies are warranted to determine the cause-consequence relationship between negative parenting style and NSSI. Third, qualitative data regarding parental styles of adolescents with NSSI may facilitate the explanations of our findings, but we did not collect such data. Future studies with prospective design, in-depth qualitative data, and representative samples from other parts of China are needed to address limitations of this study.

## Conclusion

The considerably high prevalence of NSSI, repeated NSSI, and severe NSSI in adolescents aged 10–17 years highlight the importance and pressing need of NSSI intervention in this part of China. The associations between negative parenting styles and NSSI, repeated NSSI, and severe NSSI suggest that the design of school-based prevention and education programs to prevent or reduce should consider adolescents’ negative parental styles. Promoting good parenting practices should be an essential part of such intervention programs.

## Supplemental Information

10.7717/peerj.10493/supp-1 Supplemental Information 1 rawdata.

10.7717/peerj.10493/supp-2 Supplemental Information 2 questionnaire.

10.7717/peerj.10493/supp-3 Supplemental Information 3 Questionaire in chinese.

## Additional Information and Declarations

### Data Availability

The following information was supplied regarding data availability: The raw data are available in the Supplemental File.
