# Supplementary material for: Association between parenting and non-suicidal self-injury among adolescents in Yunnan, China: a cross-sectional survey
Source: PeerJ. 2020 Dec 7;8:e10493. doi: 10.7717/peerj.10493 (PMC7727394; doi:10.7717/peerj.10493)
Supplement: Supplemental Information 2 [file peerj-08-10493-s002.docx]

| **A1** Gender | 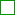 male 　　　　　　　 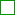 female |
| --- | --- |
| **A2** Ethnicity | 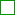 Han 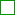 Bai 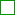 Yi 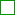 Hui 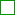 Wa  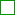 other, please fill in______________ |
| **A3** Birth date | _____________ （yyyy/mm/dd） |
| **A4** Place of residence | 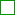 urban 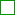 rural |
| **A6** School | Please fill in ____________________________ |
| **A7** Grade | Please fill in (primary/middle/high school)______________ |
| **A8** Do you board? | 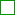 Yes 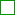 No |
| **A9** Are you the only child? | 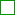 Yes 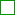 No |
| **A10** The number of transfers from elementary school to the present (not enrolling in a college) | Please fill in ：____________________________ |
| Basic situation of parents | **A12.3** Your father’s educational level ：  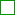 primary school and below 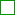 middle school  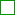 high school 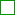 college and above 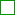 unknown |
|  | **A12.7** Your mother’s educational level ：  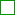 primary school and below 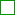 middle school  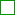 high school 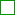 college and above 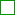 unknown |
|  | **A12.9** Your parents' marital status  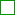in marriage 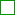divorced 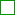remarriage 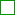windowed |
|  | **A13.2** Did your father go out to work for more than half a year (6 months) in last year? 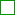 Yes 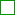 No |
|  | **A13.9** Did your mother go out to work for more than half a year (6 months) in last year? 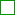 Yes 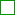 No |
| **A16** Other situations | **A16** Do you drink alcohol? (Here we define drinking as "drinking a glass of red wine, a bottle of beer, a small glass of other wine or an alcoholic beverage", excluding "sipping a sip of wine.")  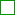 Yes 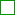 No |

| **NSSI Behaviors** | | | | | | | | | |
| --- | --- | --- | --- | --- | --- | --- | --- | --- | --- |
| ***Guidance****: Have you ever intentionally (rather than accidentally/accidentally) performed the following behavior without suicidal motivation? According to the description of "The behavior that occurred in your past life", if it exists objectively, please fill in the occurrence The approximate number of times (0 times, 1 time, 2-4 times, 5 times or more), and then fill in the degree of damage to your body by this behavior (none, mild, moderate, severe, extremely severe). Among them, "none" means no damage to the skin, "extremely severe" means that the degree of damage to the body requires hospitalization. Please mark √ in the corresponding box.* | | | | | | | | | |
| **Behaviors in your past life** | **Occurrence** | | | | **Severity** | | | | |
|  | 0 | 1 | 2-4 | >5 | None | Mild | Moderate | Severe | Extremely severe |
| **E1** Intentionally scratch your skin with glass, knife, etc. |  |  |  |  |  |  |  |  |  |
| **E2** Poked the wound to prevent the wound from healing deliberately |  |  |  |  |  |  |  |  |  |
| **E3** Burn/scald your skin with cigarette butts, lighters or other things intentionally |  |  |  |  |  |  |  |  |  |
| **E4** Intentional tattooing or patterning on the body (except for tattooing) |  |  |  |  |  |  |  |  |  |
| **E5** Scraping and bleeding your skin deliberately |  |  |  |  |  |  |  |  |  |
| **E6** Deliberately pierce things into skin or under nails |  |  |  |  |  |  |  |  |  |
| **E7** Deliberately hitting something with your head, causing bruising |  |  |  |  |  |  |  |  |  |
| **E8** Deliberately plucked his hair |  |  |  |  |  |  |  |  |  |
| **E9** Deliberately hitting hard things such as walls or glass with your hands |  |  |  |  |  |  |  |  |  |
| **E10** Intentionally violently scratching yourself to the point of being scarred or bleeding |  |  |  |  |  |  |  |  |  |
| **E11** Deliberately puncture a part of the body with a needle, nail or other thing to bleed |  |  |  |  |  |  |  |  |  |
| **E12** Deliberately rubbing the skin to bleed |  |  |  |  |  |  |  |  |  |
| **E13** Beating yourself deliberately causing bruising |  |  |  |  |  |  |  |  |  |
| **E14** Intentionally use rope or other things to strangle one's wrist and other parts |  |  |  |  |  |  |  |  |  |
| **E15** Intentionally letting others hit themselves or bite themselves to hurt their bodies |  |  |  |  |  |  |  |  |  |
| **E16** Intentionally electrocuting yourself without risk of life |  |  |  |  |  |  |  |  |  |
| **E17** Biting yourself deliberately causing skin damage |  |  |  |  |  |  |  |  |  |
| **E18** Intentionally ignite or touch the flame in your hand |  |  |  |  |  |  |  |  |  |
| **E19** If you have any other ways to intentionally harm yourself that are not mentioned in this questionnaire, please write | | | | |  | | | | |

| **Parental Rearing Style** | | | | |
| --- | --- | --- | --- | --- |
| ***Guidance****: Please recall the way your parents get along with you in daily life. Underline the option that best suits your situation.* | | | | |
| **Items** |  | **Never** | **Occasionally** | **Frequently** |
| **R1** My father/mother often lost temper with me without knowing the reason. | Father |  |  |  |
|  | Mother |  |  |  |
| **R2** Father/Mother praise me | Father |  |  |  |
|  | Mother |  |  |  |
| **R3** I hope my father/mother will not worry too much about what I am doing. | Father |  |  |  |
|  | Mother |  |  |  |
| **R4** My parents often punish me more than I should. | Father |  |  |  |
|  | Mother |  |  |  |
| **R5** My father/mother asked me to come home and have to explain to him/her what I did outside. | Father |  |  |  |
|  | Mother |  |  |  |
| **R6** I think my parents try to make my teenage life more meaningful and colorful. | Father |  |  |  |
|  | Mother |  |  |  |
| **R7** My father/mother often criticized me in front of others for being lazy and useless. | Father |  |  |  |
|  | Mother |  |  |  |
| **R8** My father/mother did not allow me to do something that other children could do because he (she) was afraid that I would have an accident. | Father |  |  |  |
|  | Mother |  |  |  |
| **R9** My father/mother always tried to encourage me and make me a leader. | Father |  |  |  |
|  | Mother |  |  |  |
| **R10** I feel that the worry of my father/mother about my possible accident is exaggerated and excessive. | Father |  |  |  |
|  | Mother |  |  |  |
| **R11** When encountering discomfort, I can feel that my father/mother is encouraging me as much as possible, so that I can be comforted. | Father |  |  |  |
|  | Mother |  |  |  |
| **R12** I am often regarded as a "scapegoat" or "black sheep" at home. | Father |  |  |  |
|  | Mother |  |  |  |
| **R13** I can receive his/her love of me through the words and emotions of my parents. | Father |  |  |  |
|  | Mother |  |  |  |
| **R14** My parents often treat me in a way that makes me embarrassed. | Father |  |  |  |
|  | Mother |  |  |  |
| **R15** My father/mother often allows me to go where I like to go without he or she being overly worried. | Father |  |  |  |
|  | Mother |  |  |  |
| **R16** I think the parent/mother interferes with anything I do. | Father |  |  |  |
|  | Mother |  |  |  |
| **R17** I feel a warm, considerate and intimate feeling with my parents. | Father |  |  |  |
|  | Mother |  |  |  |
| **R18** My father/mother has strict restrictions on what I should and should not do and never give in. | Father |  |  |  |
|  | Mother |  |  |  |
| **R19** Even a small fault, my father/mother punished me. | Father |  |  |  |
|  | Mother |  |  |  |
| **R20** My father/mother always decides what I should wear or what I should look like. | Father |  |  |  |
|  | Mother |  |  |  |
| **R21** When the things I do succeed, I feel that my parents are very proud of me. | Father |  |  |  |
|  | Mother |  |  |  |
