# Supplementary material for: Association between parenting and non-suicidal self-injury among adolescents in Yunnan, China: a cross-sectional survey
Source: PeerJ. 2020 Dec 7;8:e10493. doi: 10.7717/peerj.10493 (PMC7727394; doi:10.7717/peerj.10493)
Supplement: Supplemental Information 3 [file peerj-08-10493-s003.docx]

| **A1** 性别 | 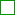 男 　　　　　　　 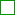 |
| --- | --- |
| **A2** 民族 | 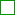 汉族 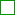 白族 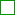 彝族 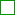 回族 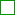 佤族  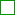 其他，请填写______________ |
| **A3** 出生日期 | _____________ （年/月/日） |
| **A4** 居住地 | 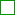 城镇 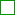 农村 |
| **A6** 学校 | 请填写 ____________________________ |
| **A7** 年级 | 请填写 (小学/初中/高中)______________ |
| **A8** 是否住校 | 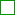 是 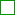 否 |
| **A9** 是否独生子女 | 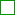 是 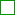 否 |
| **A10** 从小学到现在转学次数（升学不包括在内） | 请填写 ：____________________________ |
| 父母基本情况 | **A12.3** 父亲的文化程度 ：  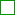 小学及以下 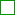 初中  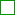 高中 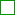 大学及以上 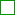 不知道 |
|  | **A12.7** 父亲的文化程度 ：  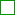 小学及以下 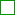 初中  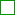 高中 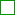 大学及以上 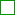 不知道 |
|  | **A12.9** 父母的婚姻状况  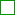 在婚 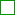 离异 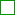 再婚 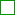 丧偶 |
|  | **A13.2** 过去一年你的父亲是否在外打工？（超过6个月） 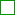 是 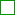 否 |
|  | **A13.9** 过去一年你的父亲是否在外打工？（超过6个月） 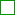 是 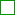 否 |
| **A16** 其他情况 | **A16** 你是否饮酒？ (这里我们将饮酒定义为“一杯红酒，一杯啤酒，一小杯其他酒或其他含酒精的饮品”，不包括“尝一口酒”。）  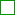 是 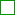 否 |

| **自伤行为** | | | | | | | | | | |
| --- | --- | --- | --- | --- | --- | --- | --- | --- | --- | --- |
| ***指导语****：你曾经在没有自杀动机的情况下，故意地(而非意外/偶然）做出过下列行为吗?根据“您过去生活中曾发生的行为”的描述，若客观存在，请填写发生的大概次数(0 次、1 次、2-4 次、5 次以上)，接着填写这一行为对您身体的伤害程度(无、轻度、中度、重度、极重)。其中，“无”代表对皮肤没有任何损伤，“极重度”是指对身体的伤害程度需要住院治疗。请您在相应格子中划√。* | | | | | | | | | | |
| **您过去生活中曾发生的行为** | **发生次数** | | | | **对身体的伤害程度** | | | | | |
|  | **0** | **1** | **2-4** | **>5** | **无** | **轻** | **中** | **重** | **极重** |  |
| **E1 故意用玻璃、小刀等划伤自己的皮肤** |  |  |  |  |  |  |  |  |  |  |
| **E2 故意戳开伤口，阻止伤口的愈合** |  |  |  |  |  |  |  |  |  |  |
| **E3 故意用烟头、打火机或其他东西烧/烫伤自己的皮肤** |  |  |  |  |  |  |  |  |  |  |
| **E4 故意在身上刺字或图案等(纹身行为除外)** |  |  |  |  |  |  |  |  |  |  |
| **E5 故意把自己的皮肤刮出血** |  |  |  |  |  |  |  |  |  |  |
| **E6 故意把东西刺入皮肤或插进指甲下** |  |  |  |  |  |  |  |  |  |  |
| **E7 故意用头撞击某物,以致出现淤伤** |  |  |  |  |  |  |  |  |  |  |
| **E8 故意拔自己的头发** |  |  |  |  |  |  |  |  |  |  |
| **E9 故意用手打墙或玻璃等较硬的东西** |  |  |  |  |  |  |  |  |  |  |
| **E10 故意猛烈的乱抓自己,达到了有伤痕或者流血的程度** |  |  |  |  |  |  |  |  |  |  |
| **E11 故意用针、钉子或其它东西把身体某一个部分扎出血** |  |  |  |  |  |  |  |  |  |  |
| **E12 故意把皮肤擦出血** |  |  |  |  |  |  |  |  |  |  |
| **E13 故意捶打自己以致出现淤伤** |  |  |  |  |  |  |  |  |  |  |
| **E14 故意用绳子或其它东西勒自己的手腕等部位** |  |  |  |  |  |  |  |  |  |  |
| **E15 故意让他人打自己或者咬自己,以此伤害自己的身体** |  |  |  |  |  |  |  |  |  |  |
| **E16 故意在没有生命危险情况下让自己触电** |  |  |  |  |  |  |  |  |  |  |
| **E17 故意咬自己以致皮肤破损** |  |  |  |  |  |  |  |  |  |  |
| **E18 故意在手里点火或触摸火焰** |  |  |  |  |  |  |  |  |  |  |
| **E19 若你还有哪些故意伤害自己的方式没有有在此问卷中提及，请写出** |  | | | | | | | | | |

| **父母教养方式** |
| --- |

| ***指导语：以下请您回想父母在日常生活中与你的相处方式，请在最符合您情况的选项下划√。*** | | | | | |
| --- | --- | --- | --- | --- | --- |
| **项 目** |  | **从不** | **偶尔** | **经常** | **总是** |
| **R1** 父/母亲常常在我不知道原因的情况下对我大发脾气。 | 父 |  |  |  |  |
|  | 母 |  |  |  |  |
| **R2** 父/母亲赞美我。 | 父 |  |  |  |  |
|  | 母 |  |  |  |  |
| **R3** 我希望父/母亲对我正在做的事不要过分担心。 | 父 |  |  |  |  |
|  | 母 |  |  |  |  |
| **R4** 父/母亲对我的惩罚往往超过我应受的程度。 | 父 |  |  |  |  |
|  | 母 |  |  |  |  |
| **R5** 父/母亲要求我回到家里必须得向他/她说明我在外面做了什么事。 | 父 |  |  |  |  |
|  | 母 |  |  |  |  |
| **R6** 我觉得父/母亲尽量使我的青少年时期的生活更有意义和丰富多彩。 | 父 |  |  |  |  |
|  | 母 |  |  |  |  |
| **R7** 父/母亲经常当着别人的面批评我既懒惰又无用。 | 父 |  |  |  |  |
|  | 母 |  |  |  |  |
| **R8** 父/母亲不允许我做一些其他孩子可以做的事情,因为他（她）害怕我会出事。 | 父 |  |  |  |  |
|  | 母 |  |  |  |  |
| **R9** 父/母亲总试图鼓励我,使我成为佼佼者。 | 父 |  |  |  |  |
|  | 母 |  |  |  |  |
| **R10** 我觉得父/母亲对我可能出事的担心是夸大的、过分的。 | 父 |  |  |  |  |
|  | 母 |  |  |  |  |
| **R11** 当遇到不顺心的事时,我能感到父/母亲在尽量鼓励我,使我得到安慰。 | 父 |  |  |  |  |
|  | 母 |  |  |  |  |
| **R12** 我在家里往往被当作“替罪羊”或“害群之马”。 | 父 |  |  |  |  |
|  | 母 |  |  |  |  |
| **R13** 我能通过父/母亲的言谈、表情感受到他（她）很喜欢我。 | 父 |  |  |  |  |
|  | 母 |  |  |  |  |
| **R14** 父/母亲常以一种使我很难堪的方式对待我。 | 父 |  |  |  |  |
|  | 母 |  |  |  |  |
| **R15** 父/母亲常常允许我到我喜欢去的地方,而他（她）又不会过分担心。 | 父 |  |  |  |  |
|  | 母 |  |  |  |  |
| **R16** 我觉得父/母亲干涉我做的任何一件事。 | 父 |  |  |  |  |
|  | 母 |  |  |  |  |
| **R17** 我觉得与父/母亲之间存在一种温暖、体贴和亲热的感觉。 | 父 |  |  |  |  |
|  | 母 |  |  |  |  |
| **R18** 父/母亲对我该做什么、不该做什么都有严格的限制而且绝不让步。 | 父 |  |  |  |  |
|  | 母 |  |  |  |  |
| **R19** 即使是很小的过错，父/母亲也惩罚我。 | 父 |  |  |  |  |
|  | 母 |  |  |  |  |
| **R20** 父/母亲总是左右我该穿什么衣服或该打扮成什么样子。 | 父 |  |  |  |  |
|  | 母 |  |  |  |  |
| **R21** 当我做的事情取得成功时,我觉得父/母亲很为我自豪。 | 父 |  |  |  |  |
|  | 母 |  |  |  |  |
